# Assessment of a novel computer software in diagnosing radiocarpal osteoarthritis on plain radiographs of patients with previous distal radius fracture

**DOI:** 10.1016/j.ocarto.2020.100112

**Published:** 2020-10-28

**Authors:** Muhanned Ali, Elisabeth Brogren, Isam Atroshi

**Affiliations:** a Department of Orthopedics, Kristianstad and Hässleholm Hospitals, Hässleholm, Sweden; b Department of Hand Surgery, Skåne University Hospital, Malmö, Sweden; c Department of Clinical Sciences - Orthopedics, Lund University, Lund, Sweden

**Keywords:** Radiocarpal osteoarthritis, Artificial intelligence, Diagnostic study, Distal radius fracture

## Abstract

**Objective:**

Osteoarthritis (OA) has primarily been diagnosed with plain radiographs assessed visually by examiners with regard to joint space width and presence of subchondral sclerosis, cysts and osteophytes. The increasing use of artificial intelligence has seen software developed to examine plain radiographs for diagnosing OA, based on observed OA-associated subchondral bone microarchitecture changes. A software for computerized texture analysis has been developed to identify knee OA. The aim of this study was to assess the software's ability to identify radiocarpal OA.

**Design:**

Presence of radiocarpal OA on 63 wrist radiographs of patients with a previous distal radius fracture was assessed independently by two surgeons experienced in assessing radiographs, and classified according to Kellgren-Lawrence (38 OA, 25 no OA). First, the computer software, not previously trained to identify wrist OA, assessed presence of radiocarpal OA on the 63 radiographs. In a second step, 144 labeled wrist radiographs with and without radiocarpal OA was used to train the computer software. Presence of OA on the original 63 radiographs were then reassessed by the software. Sensitivity, specificity and area under the curve (AUC) were calculated to determine the software's ability to discriminate between cases with and without OA.

**Results:**

Before training, sensitivity was 76% (95% CI 59–88), specificity 25% (10–47), and AUC 0.50 (0.35–0.65). After training, sensitivity was 46% (29–63), specificity 70% (47–87), and AUC 0.58 (0.43–0.73).

**Conclusion:**

The software for computerized texture analysis of subchondral bone developed to identify knee OA could not detect OA of the radiocarpal joint.

## 1 Introduction

Osteoarthritis (OA) has primarily been diagnosed with plain radiographs assessed visually by examiners with regard to joint space width and presence of subchondral sclerosis, cysts and osteophytes. Besides being time-consuming, assessment of plain radiographs for OA may not be sensitive enough in detecting early OA. Artificial intelligence is being increasingly used in medicine, including applications for diagnostic purposes. It has been proposed that changes in the microarchitecture of subchondral bone can be seen in early OA, before cartilage degradation and subsequent joint space narrowing are visualized on radiographs [1,2]. Computerized fractal-based algorithms that assess the orderliness of the subchondral trabecular bone have been developed [[3], [4], [5], [6]]. These models are based on the observations that trabecular bone has fractal properties (i.e. it is too irregular to be described in traditional geometrical language and is self-similar over a range of scales) and that 2-dimensional radiographs provide information of the underlying 3-dimensional texture of the trabecular bone structure [7,8]. One such computerized texture algorithm (Image biopsy lab©, Vienna) has been shown to be capable of distinguishing knee joints with OA from those without OA on plain radiographs with an accuracy of 84% [4]. The use of this software has been reported to increase the consistency between physicians when grading radiographic features of OA [9]. Radiocarpal OA is a known long-term complication after wrist trauma such as distal radius fracture [10] and, although less common than in big joints, primary OA may involve the radiocarpal joint. A software that can detect OA in the wrist joint in a standardized manner with good accuracy can be of benefit in clinical research about wrist OA. The novel computer software developed by (Image biopsy lab©, Vienna) for knee OA, based on the changes seen in the microarchitecture of the subchondral bone, has not been assessed with regard to OA in the wrist joint. A widely used classification to grade OA is the Kellgren-Lawrence system based on joint space width and presence of subchondral sclerosis, cysts and osteophytes [11]. These changes are seen in OA affecting various joints and the classification is used to identify OA in different joints. We hypothesized that the changes in the subchondral bone in OA would be similar in different joints and that the software shown to detect knee OA would be able to identify radiocarpal OA. This study aimed to assess the ability of the computer software to diagnose radiocarpal OA in patients with a previous distal radius fracture.

## 2 Materials and methods

This study included patients who had participated in a prospective cohort study of patients with acute distal radius fracture conducted 2001–2002, in northeast Skåne in Sweden [12]. From October 2014 through April 2015 (12–14 years after the original study), 63 patients treated with casting, closed reduction and casting or closed reduction and percutaneous surgical fixation, attended a radiographic examination. Radiocarpal OA was classified according to Kellgren-Lawrence (5 categories ranging from 0 [no OA] to 4 [severe OA]) [11], independently by an orthopedic surgeon (MA) and a hand surgeon (EB). In 12 patients (19%), there were discrepancies regarding the presence or absence of OA, which were resolved by discussion and consensus. Of the 63 patients (Table 1), 25 were classified as having no radiocarpal OA (grade 0) and 38 as having radiocarpal OA (grade 1 or higher) [10]. The study was approved by The Ethical Review Board of Lund University.

**Table 1:** Characteristics of patients with a follow-up of 12–14 years after distal radius fracture.

	Radiocarpal OA n ​= ​38	No radiocarpal OA n ​= ​25
Age at fracture, mean (SD) y	53 (8)	53 (10)
Women, n (%)	27 (71)	20 (80)
Fracture involved dominant hand, n (%)	18 (47)	9 (36)
Surgical treatment^a^, n (%)	17 (45)	7 (28)

a Closed reduction and external fixation or percutaneous pinning. Nine cases were fixed using pins in the subchondral bone. Pins were routinely extracted 4–6 weeks after surgery.

**Figure 1:**
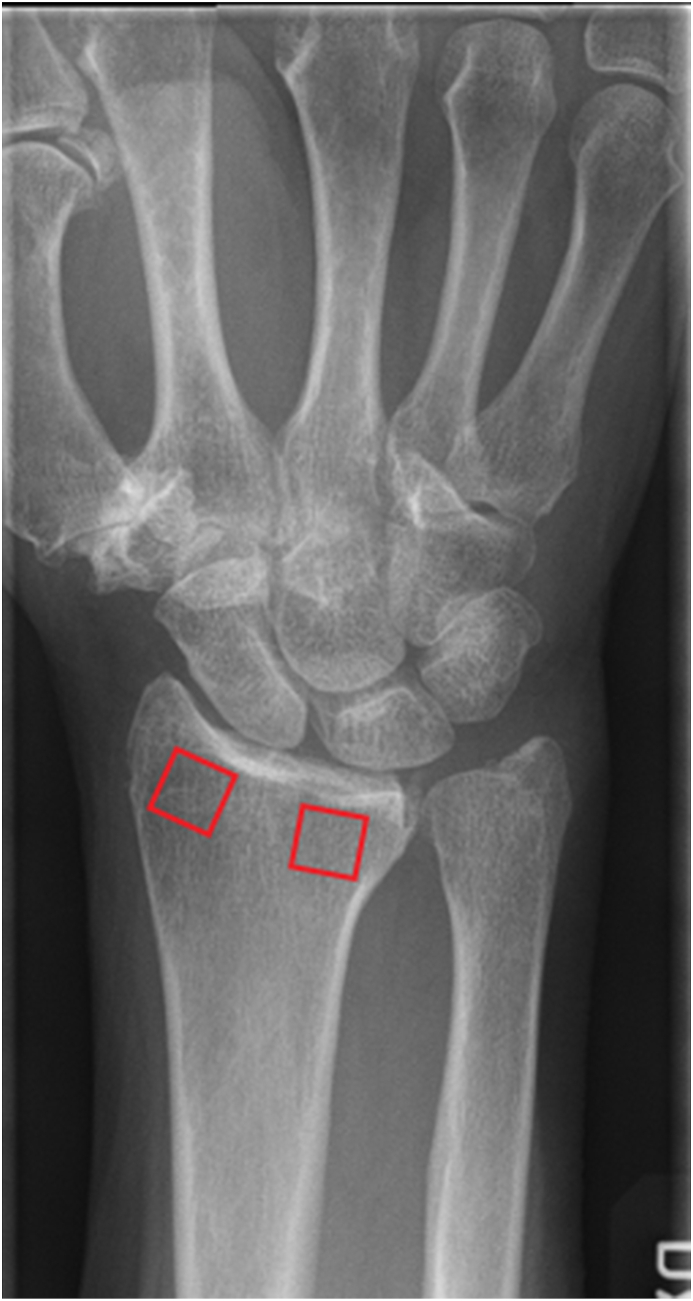
Radiograph of the wrist with the 2 selected regions of interest (ROI) marked (red squares). Visual assessment of radiocarpal osteoarthritis used the Kellgren-Lawrence classification (4 grades: 0, no osteoarthritic changes; 1, possible sclerosis and minimal osteophytes without joint space narrowing; 2, slight joint space narrowing and osteophytes; 3, moderate joint space narrowing, osteophytes, and possible cyst formation; 4, severe joint space narrowing, osteophytes, and sclerosis).

The computer software (Image biopsy lab©) is an automated system that analyzes the subchondral trabecular bone on plain radiographs in two rectangular bone regions, called regions of interest (ROIs), for detection of OA (Fig. 1). The placement of the ROIs at the subchondral bone of the distal radius radiographs was performed using the proprietary software IB Lab TX Analyzer and was consistent across the cases.

In a first step, the computer software (previously trained to identify knee OA) assessed the radiographs of the 63 study patients for detection of radiocarpal OA. Two radiographs could not be assessed by the software due to technical issues. Thus 61 radiographs were used in the analyses. In a second step and in order to train the computer software to detect radiocarpal OA, we used 196 consecutive wrist radiographs (37 bilateral) for patients attending the orthopedic department for any reason. These radiographs were examined and graded according to the Kellgren-Lawrence classification independently by the two surgeons (MA and EB). In 164 (84%) of the radiographs (28 bilateral), the surgeons agreed on the classification and these radiographs (75 OA and 89 no OA) were used as a labeled dataset to train the software to recognize radiocarpal OA. According to the software's developers a sample size of 75 radiographs per group (OA and no OA) would be needed for this purpose. Of the 164 radiographs, 20 could not be used due to technical issues, leaving 144 radiographs that were used for training the software. In a third step, the trained computer software reassessed the radiographs of the 63 patients with previous distal radius fracture. In this analysis 58 of the radiographs could be reassessed.

## 3 Analyses

Sensitivity, specificity and the area under the curve (AUC) were calculated before and after training to determine the computer software's ability to distinguish between OA and non-OA wrists.

## 4 Results

Before training the software, sensitivity was 76% (95% CI 59–88), specificity 25% (10–47), and AUC 0.50 (0.35–0.65), indicating that the computer software had poor ability to discriminate between radiocarpal OA and no OA. After training, sensitivity was 46% (95% CI 29–63), specificity 70% (47–87), and AUC 0.58 (0.43–0.73), implying low ability to improve the performance through training with a labeled data set. Using only the 51 radiographs of the original 63 patients, in which the two surgeons made identical first-time assessment regarding the presence of radiocarpal OA gave similar results.

## 5 Discussion

In contrast to prior successful results of computerized textural analysis of knee joint OA, we could not reproduce the results for radiocarpal OA in patients with a previous distal radius fracture. There are several possible explanations to the poor performance of the software in this cohort. First, the computer software was developed for the knee joint and we assessed the radiocarpal joint. The mechanical and anatomical differences between the complex wrist joint and the weight-bearing knee joint may have importance in the development of OA [2]. In cross-sectional and longitudinal studies of hand and knee OA, Buckland-Wright et al. have shown that although changes were similar in early OA, with progression of the disease, cortical plate thickness in hand OA increased in 60% of the patients but did not change in knee OA patients until severe narrowing of the joint space occurred [2]. Additionally, in bone texture analyses, when the methods developed for detection of knee OA were directly applied to the finger joints, the results were considered encouraging, but not as reliable as the results shown for knee OA. This was due to the fact that the selected ROIs on hand radiographs were considerably smaller than those on knee radiographs rendering the data not enough for analysis [3]. Altogether, those findings may suggest some differences in OA pathology in the hand compared to the knee that may affect texture analyses.

A second possible explanation is that patients with fracture might have altered microstructure of the subchondral bone. Rozental et al. showed worse trabecular bone microarchitecture at the distal radius manifested by reduced trabecular plate volume, number, thickness and connectivity three months after a distal radius fracture in 40 premenopausal women compared to 80 non-fracture controls [13]. In fact, altered microstructure of the subchondral bone has been shown long time after a fracture. Stein et al. found a deterioration of the trabecular microarchitecture at the distal radius in 68 postmenopausal women at a mean of 5.5 (SD 5.6) years after a variety of central and peripheral fragility fracture, including distal radius fracture, compared to 101 postmenopausal women without history of a fragility fracture [14]. Furthermore, Sornay-Rendu et al. found similar architectural alterations of the trabecular and cortical bone in 101 postmenopausal women 13 years after a fragility fracture, compared to 101 matched controls who never had a fracture [15]. It is possible that longstanding alterations of the trabecular bone after fragility fractures affect the computer software ability to differentiate cases with OA from those without OA.

The results showed that the sensitivity worsened after training the computer software while the specificity improved substantially, however, without improvement in the overall performance as the AUC was essentially unchanged. We have no explanation to this shift in sensitivity and specificity. The person who operated the software was blinded to the labeling of the original 63 radiographs throughout the study, both before and after the software training.

Our study has limitations. The labeled radiographs used to train the computer to recognize radiocarpal OA comprised patients with mixed genesis to the OA, including distal radius fracture, scaphoid non-union, scapholunate ligament injury and Kienböcks disease. Different pathogenesis to the OA in this group could have affected the computer software's ability to train and the results may have been different if the software were trained with only cases of radiocarpal OA secondary to distal radius fracture. Another limitation was that we used the Kellgren-Lawrence scale as golden standard for defining OA. The scale is based on the presence of osteophytes; i.e. joint space narrowing, subchondral sclerosis and deformity are considered insufficient changes unless seen in conjunction with osteophytes [11]. Since the computer software is designed to recognize alterations in the subchondral bone rather than recognizing osteophytes, this could have affected our results. However, it is the most common classification system and the radiographs were examined independently by two surgeons and the grading was the same for the majority.

Nine of the 63 study patients were treated using pins in the subchondral bone area of the distal radius which might have affected OA analysis. However, these pins were routinely extracted 4–6 weeks after surgery and the assessment of OA was done 12–14 years after the fracture and therefore, we believe it is unlikely that initial treatment with pins would affect the results. Another limitation is that the size of the sample used in our study may have been relatively small. However, given the results, it is uncertain whether a larger sample size can substantially improve the performance of the software.

In conclusions, the software for computerized texture analysis of the subchondral bone developed for detection of knee OA could not detect OA of the radiocarpal joint. The use of artificial intelligence as a diagnostic tool in OA should be subject to careful evaluation.

## Credit author statement

**Muhanned Ali:** Conceptualization, Methodology, Formal Analysis, Investigation, Resources, Data Curation, Writing – Original Draft, Writing – Review & Editing, Visualization, Project Administration, Funding Acquisition **Elisabeth Brogren:** Conceptualization, Methodology, Investigation, Writing – Original Draft, Visualization **Isam Atroshi:** Conceptualization, Methodology, Formal Analysis, Investigation, Resources, Writing – Original Draft, Writing – Review & Editing, Visualization, Supervision, Project Administration.

## Author contributions

All authors have made a substantial contribution to the conception and design of this study, interpretation of the data, drafting and revising the content of this article, and approved the final submitted version. In addition, MA and EB have collected the reported data and MA and IA have done the analyses and take full responsibility for the integrity of the work presented.

## Funding source

The study was supported by an internal research grant from Region Skåne (FoU research grant). There was no source of external funding for this study.

## Declaration of competing interest

Muhanned Ali, Elisabeth Brogren and Isam Atroshi declare that they have no conflict of interest.
